# Bibliometric analysis of stem cells for spinal cord injury: current status and emerging frontiers

**DOI:** 10.3389/fphar.2023.1235324

**Published:** 2023-07-18

**Authors:** Zhizhong Shang, Pingping Wanyan, Mingchuan Wang, Baolin Zhang, Xiaoqian Cui, Xin Wang

**Affiliations:** ^1^ The First Clinical Medical College of Lanzhou University, Lanzhou, China; ^2^ Department of Pathology and Pathophysiology, School of Basic Medicine, Gansu University of Traditional Chinese Medicine, Lanzhou, China; ^3^ Department of Nephrology, The Second Hospital of Lanzhou University, Lanzhou, China; ^4^ Chengren Institute of Traditional Chinese Medicine, Lanzhou, Gansu, China; ^5^ Department of Spine, Changzheng Hospital, Naval Medical University, Shanghai, China

**Keywords:** spinal cord injury, stem cell, bibliometric analysis, current status, emerging frontiers

## Abstract

**Background:** This study aimed to conduct a bibliometric analysis of the literature on stem cell therapy for spinal cord injury to visualize the research status, identify hotspots, and explore the development trends in this field.

**Methods:** We searched the Web of Science Core Collection database using relevant keywords (“stem cells” and “spinal cord injury”) and retrieved the published literature between 2000 and 2022. Data such as journal title, author information, institutional affiliation, country, and keywords were extracted. Afterwards, we performed bibliometric analysis of the retrieved data using Bibliometrix, VOSviewer, and CiteSpace.

**Results:** A total of 5375 articles related to stem cell therapy for spinal cord injury were retrieved, and both the annual publication volume and the cumulative publication volume showed an upward trend. neural regeneration research was the journal with the most publications and the fastest cumulative publication growth (162 articles), Okano Hideyuki was the author with the highest number of publications and citations (114 articles), Sun Yat-sen University was the institution with the highest number of publications (420 articles), and China was the country with the highest number of publications (5357 articles). However, different authors, institutions, and countries need to enhance their cooperation in order to promote the generation of significant academic achievements. Current research in this field has focused on stem cell transplantation, neural regeneration, motor function recovery, exosomes, and tissue engineering. Meanwhile, future research directions are primarily concerned with the molecular mechanisms, safety, clinical trials, exosomes, scaffolds, hydrogels, and inflammatory responses of stem cell therapy for spinal cord injuries.

**Conclusion:** In summary, this study provided a comprehensive analysis of the current research status and frontiers of stem cell therapy for spinal cord injury. The findings provide a foundation for future research and clinical translation efforts of stem cell therapy in this field.

## 1 Introduction

Spinal cord injury can lead to permanent loss of sensory and motor function, abnormal reflex activity, and autonomic nervous system dysfunction, as well as various complications such as lung infections, urinary tract infections and stones, pressure ulcers, deep vein thrombosis and pulmonary embolism, joint contractures, and heterotopic ossification (Jain et al., 2015; Ahuja et al., 2017a). There are currently over 27 million cases of spinal cord injury, with nearly 1 million new cases annually (GBD, 2016 Traumatic Brain Injury and Spinal Cord Injury Collaborators, 2019). Spinal cord injury and its complications cause severe physical and psychological suffering and economic burden to patients. Although timely and effective symptomatic treatment and care have reduced the mortality rate of SCI patients, the recovery of nerve function remains a long-term and arduous task, and currently there are no effective measures to improve the prognosis of spinal cord injury patients (Hachem et al., 2017; Donovan and Kirshblum, 2018; Bloom et al., 2020).

In the past two decades, the development of stem cells and regenerative medicine, as well as in-depth research on the pathophysiology of spinal cord injury, has brought new hope for its treatment (Shang et al., 2022a; Fan et al., 2022). Stem cells from different tissue sources can protect and regenerate the damaged spinal cord by promoting angiogenesis, immunomodulation, anti-inflammatory, anti-apoptotic, and other mechanisms, making them the most promising treatment (Ashammakhi et al., 2019; Freyermuth-Trujillo et al., 2022). Although preclinical studies have long confirmed the effectiveness of stem cell therapy, the clinical translation of stem cell therapy remains challenging. The optimal type of stem cells, source (autologous or allogeneic), transplant dose, number of transplants, transplant route (arterial, venous, intraluminal), and timing of transplantation (acute, subacute, chronic) are unclear, and stem cell transplantation may cause many adverse reactions (Shang et al., 2022a; Shang et al., 2022b). In summary, there are still many issues regarding stem cell therapy for spinal cord injury that need to be resolved.

Currently, there are numerous studies exploring stem cell therapy for spinal cord injury, laying the foundation for the clinical translation of stem cell therapy. However, in the vast literature, which articles have significant value, which countries, institutions, and authors have made important contributions, what are the current research hotspots in the field, and what are the future development directions of this field, all need to be considered and answered. So far, numerous literature reviews and expert opinions have attempted to elucidate the research status and frontiers of stem cell therapy for spinal cord injury (Tashiro et al., 2021; Lee et al., 2022; Zipser et al., 2022). However, these studies are relatively fragmented and subjectively focused, with insufficient comprehensiveness and systematicness, and less objective and quantitative, which is not conducive to researchers’ overall understanding of the field. Bibliometrics, as a method of quantitatively summarizing multidimensional information in a certain field, can help researchers grasp the research status and predict future research hotspots in a short period of time by using visualization and network-related technologies to explore research trends in a certain field (Lariviere et al., 2013; Cooper, 2015). Therefore, this study aims to use bibliometric methods to explore the research status and frontiers of stem cell therapy for spinal cord injury.

## 2 Methods

### 2.1 Data sources

This study conducted a search in the Web of Science Core Collection (WoSCC) database. The search terms used were as follows: (“spinal cord injury” OR “spinal cord injuries” OR “spinal injury” OR “spinal injuries” OR “spinal cord trauma” OR “spinal cord transection” OR “spinal cord laceration” OR “post-traumatic myelopathy” OR “spinal cord contusion")) AND (“stem cells” OR “stem cell” OR “Progenitor Cells” OR “Progenitor Cell” OR “Mother Cells” OR “Mother Cell”). The search period was from 1 January 2000 to 18 May 2023. The included document types were original research and review articles, while conference abstracts, letters, case reports, and non-English documents were excluded. The full records of the articles were obtained, including titles, publication years, authors, countries (regions), research institutions, journal names, keywords, and abstracts. As this study is a bibliometric study and did not involve human or animal participants, ethical approval was not required.

### 2.2 Research methods

This study used the Bibliometrix package in R 4.2.1 for publication volume statistics and journal source analysis. VOSviewer 1.6.18 and Scimago Graphica were used for country cooperation analysis. Citespace 6.2.R2 was used for author and institution cooperation analysis, as well as co-occurrence, clustering, growth and burst analysis of keywords.

## 3 Results

### 3.1 Publishing trend

**FIGURE 1:**
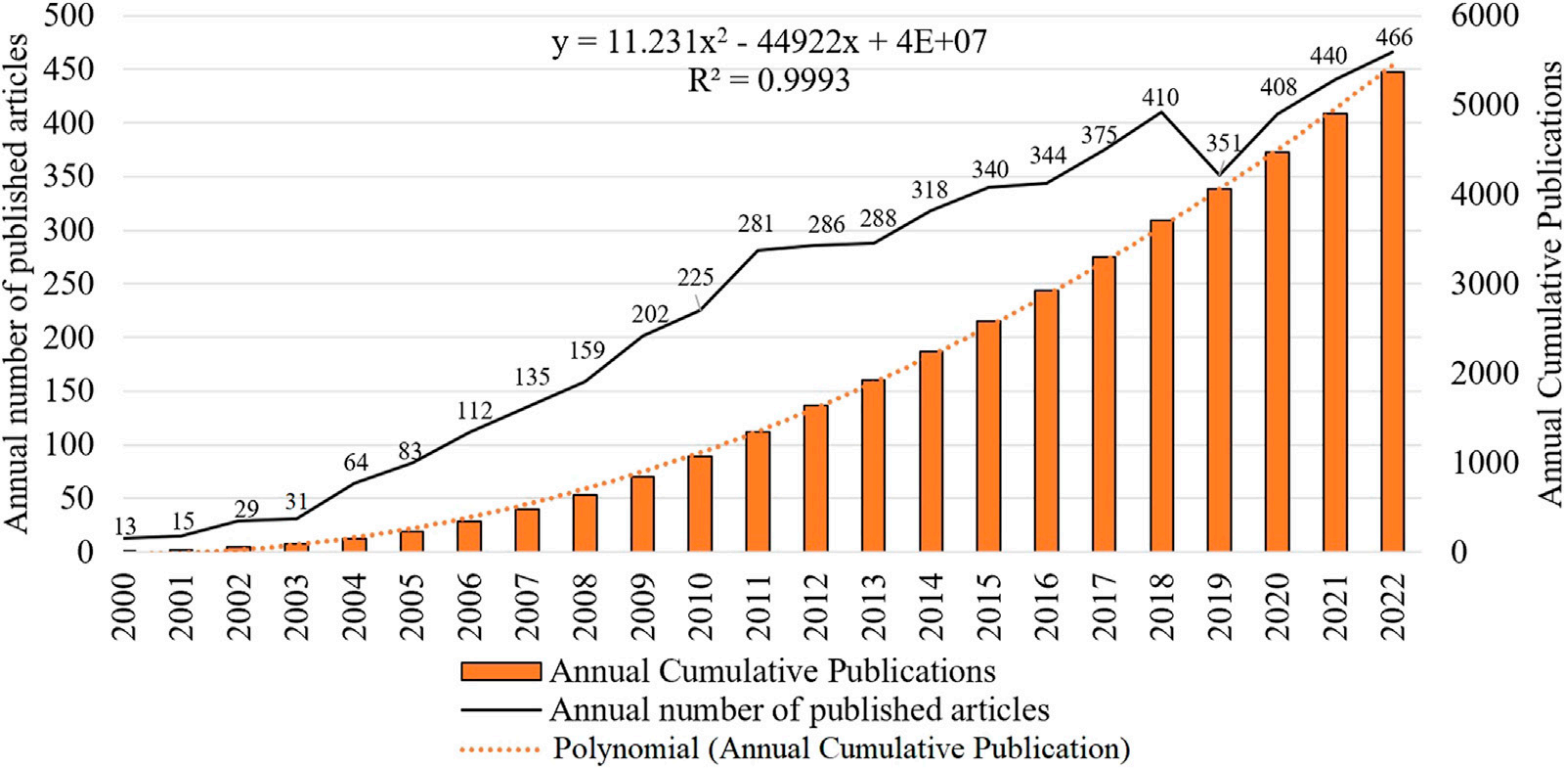
Annual publication volume and annual cumulative publication volume.

By analyzing the publication time and trend distribution of literature, the development speed and attention of stem cell therapy for spinal cord injuries in the academic community can be judged more intuitively. During the period from 2000 to 2022, a total of 5375 studies related to stem cell therapy for spinal cord injuries have been published, and the cumulative and annual publication volumes showed an overall upward trend, indicating that the research topic is receiving increasing attention from scholars and the research interest is constantly rising (Figure 1). Specifically, the annual publication volume from 2000 to 2011 showed a rapid upward trend, while from 2011 to 2018, it increased slowly but remained at a relatively high level. In 2019, there was a slight decline, followed by an upward trend, reaching its peak in 2022 with 456 publications.

### 3.2 Source journal of literature

**FIGURE 2:**
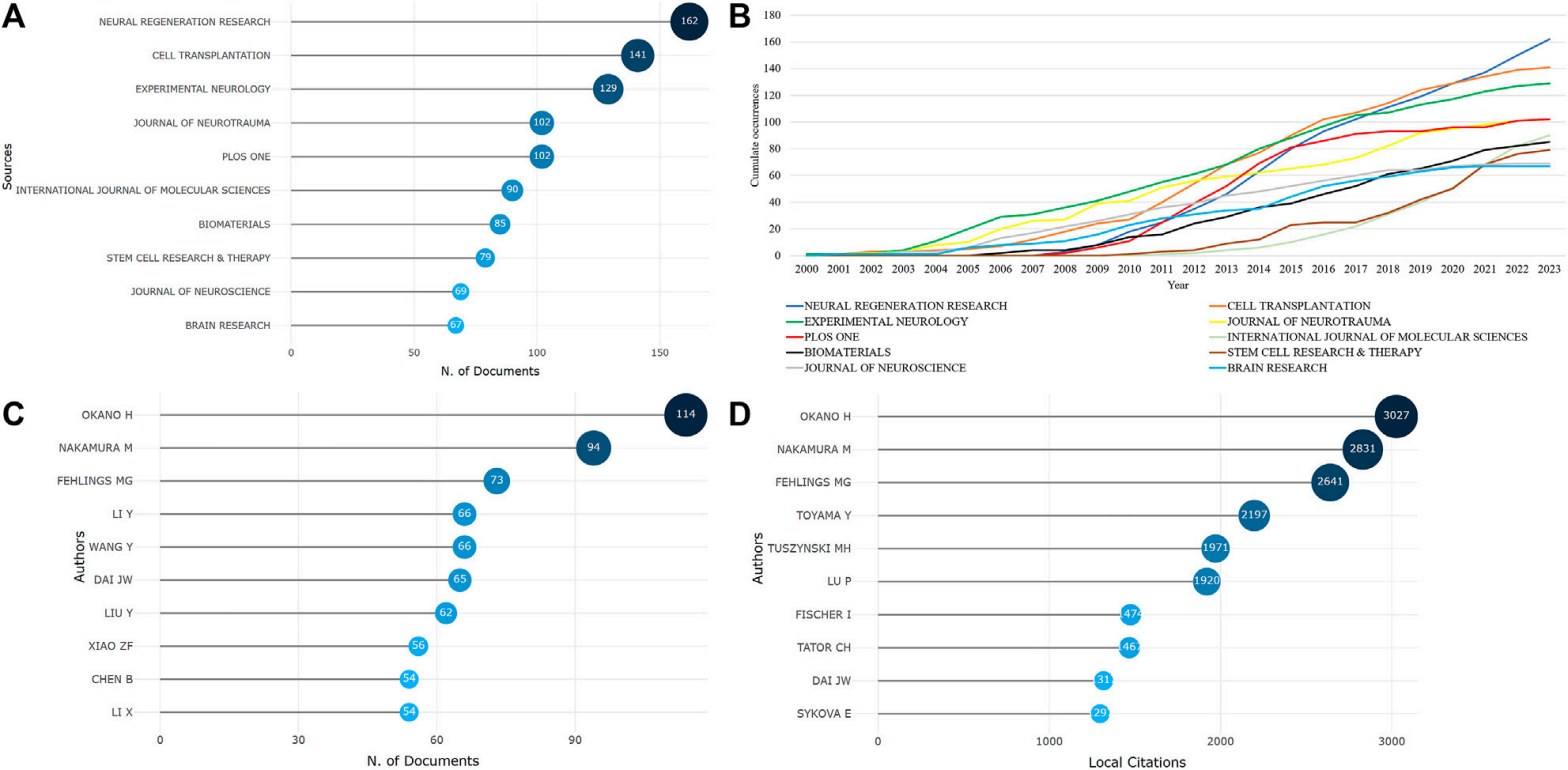
The sources of literature and author publication volume are as follows [**(A)**. Top 10 journals by publication volume; **(B)**. Top 10 journals by cumulative publication growth; **(C)**. Top 10 authors by publication volume; **(D)**. Top 10 most cited authors].

The sources of the included literature were counted, and it was found that stem cell therapy for spinal cord injury has been published in 1,081 journals since 2000. The top 10 journals with the highest publication counts are shown in Figure 2A, accounting for approximately 18.51% of the total publication volume. neural regeneration research, cell transplantation, experimental neurology, plos one, and journal of neurotrauma have published over 100 articles each. Figure 2B; Supplementary Table S1 show the top 10 journals with the fastest growth in cumulative publication volume as the years increase, with neural regeneration research showing the fastest growth rate, indicating that this journal is rapidly developing in the current field.

### 3.3 Author collaboration analysis

**FIGURE 3:**
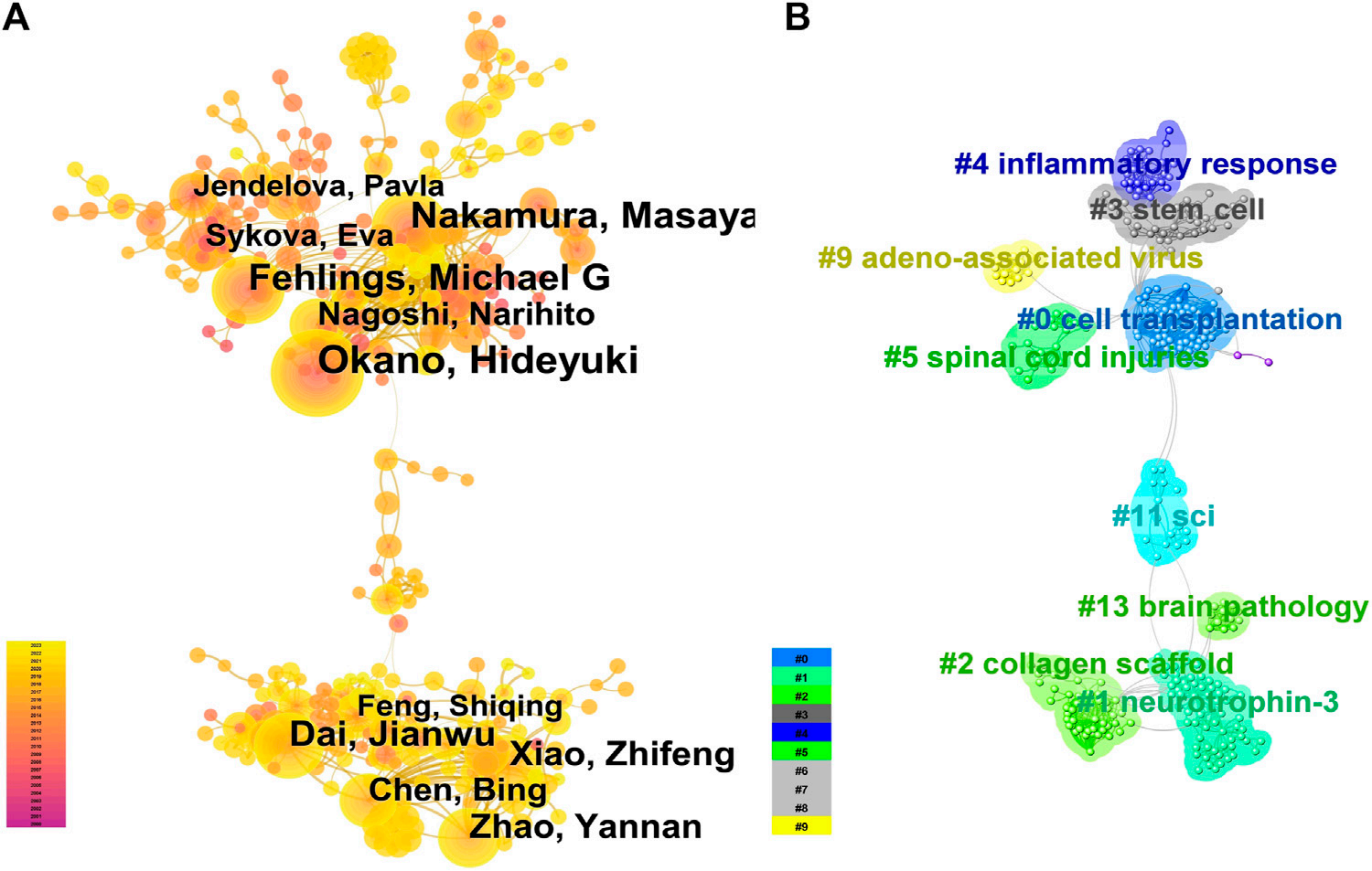
Author collaboration **(A)**. The co-occurrence analysis of authors is represented by a network graph where the size of the circle indicates the number of publications, the more connections indicate more collaborations, and the lighter colors indicate more recent publications. **(B)**. The author cluster analysis is represented by a network graph where authors with similar research interests or collaboration patterns are grouped together, and the same color represents one cluster].

A total of 19,092 authors participated in the writing and publication of related literature, with the frequency of author publication ranging from 1 to 114 publications. Most of the authors only participated in the publication of one article, accounting for 71.59% (13,668/19,092). The top three authors by publication volume were Okano Hideyuki (114 publications), Nakamura Masaya (94 publications), and Fehlings Michael G (73 publications) (Figure 2C), and they were also the most cited authors (Figure 2D). Co-occurrence analysis of authors revealed that the current research field has formed many academic groups. Okano Hideyuki, Nakamura Masaya, Sykova Eva, Xiao Zhifeng, Dai Jianwu, Zeng Yuanshan, Sahib Seaab, and others have formed different academic groups (Figure 3A). Authors within the same group have closer collaborations, but cooperation between different academic groups is insufficient. In addition, the betweenness centrality is used to measure the probability that a node is located in the shortest path between any other two points. The greater the betweenness centrality, the more important the node is. The betweenness centrality of different authors is 0, indicating that the research in this field has not yet formed a widely connected core author network. Further cluster analysis revealed that academic groups centered on Okano Hideyuki, Nakamura Masaya, Sykova Eva mostly focus on the molecular mechanisms of stem cell therapy for spinal cord injury and cell transplantation, while academic groups centered on Xiao Zhifeng, Dai Jianwu, Zeng Yuanshan, Sahib Seaab, place more emphasis on the application of biomaterials in spinal cord injury (Figure 3B).

### 3.4 Institutional cooperation analysis

**FIGURE 4:**
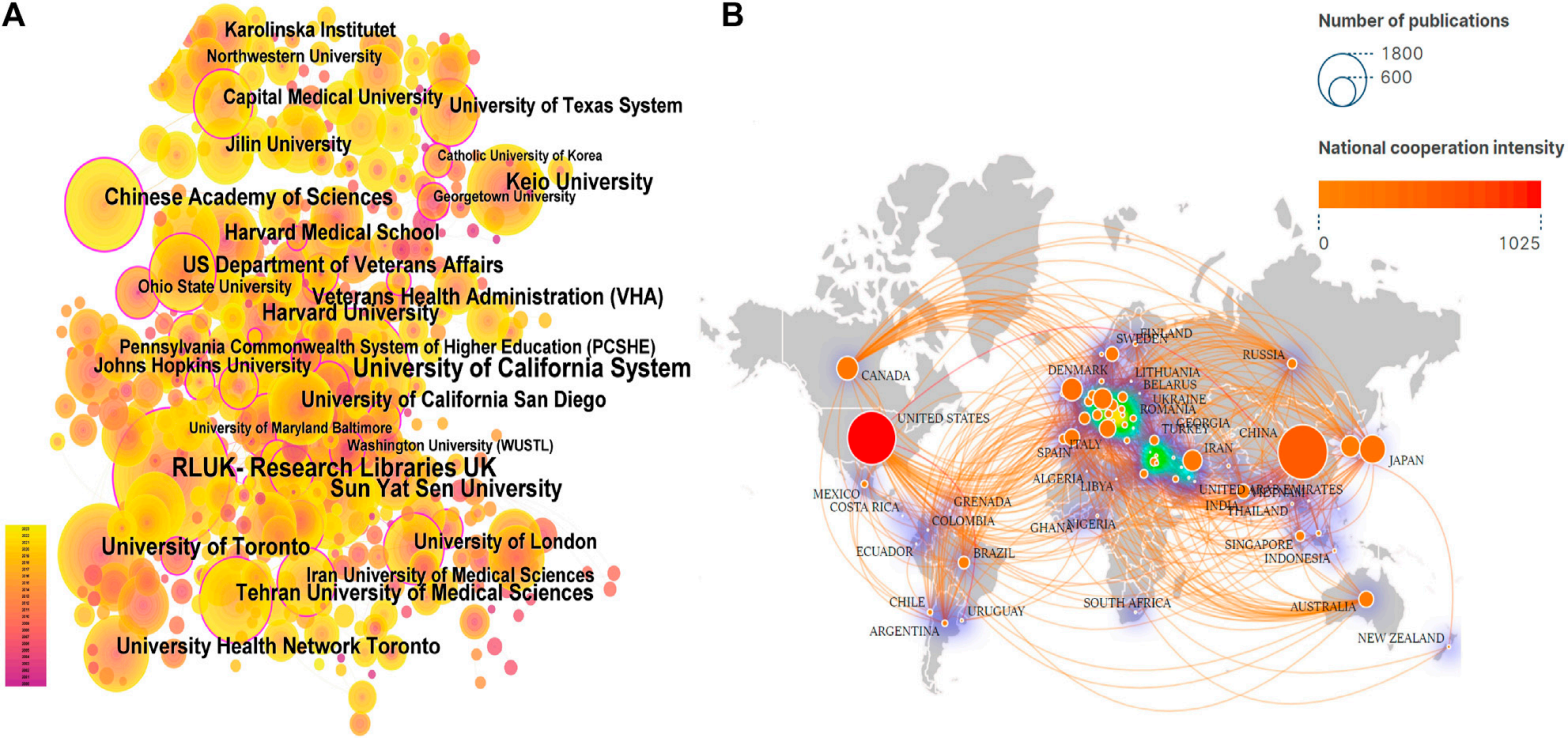
Institutional cooperation and Country Cooperation **(A)**. In institutional cooperation, the larger the circle is, the more articles are published, the more lines are connected, the more cooperation is, the lighter the color is, the later the post is, and the circle with purple outer circle represents greater intermediary centrality. **(B)**. In national cooperation, the larger the circle, the more papers the country sends, the more connections, the more cooperation between the country and other countries, the darker the color of the circle, the greater the intensity of cooperation, and the greater the density around the ring, the more the country is in the center of the research.

The analysis of the scale and degree of collaboration between institutions can provide references for the development of stem cell therapy for spinal cord injury. A total of 4,020 institutions participated in the publication of the research articles, among which Sun Yat-sen University (420 articles), University of Toronto (303 articles), and Keio University (296 articles) ranked the top three in terms of publication volume. For details on the top 10 institutions in terms of publication volume, please refer to Supplementary Table S2. However, co-occurrence analysis of the institutions revealed that although the above three institutions had the highest publication volume, their collaborations with other institutions were relatively few. On the contrary, institutions such as the RLUK-Research Libraries United Kingdom, University of California System and Harvard University had more extensive collaborations with other institutions (Figure 4A), and they had a greater influence in the field (institutions with a purple outer circle represent having a larger betweenness centrality). The top 10 institutions with betweenness centrality are shown in Supplementary Table S3.

### 3.5 Country cooperation analysis

A total of 70 countries have published literature on stem cell therapy for spinal cord injury, with China (5357 articles), the United States (4266 articles), and Japan (1370 articles) ranking the top three in terms of publication volume, see Supplementary Table S4 for details. With the globalization of knowledge and technology, collaborations between different countries are very close, with the United States having the highest level of national cooperation, indicating that the United States collaborates extensively with other countries; China has the highest publication volume, but its cooperation intensity with other countries is second to that of the United States. From the density around the circle, it can be seen that Europe is the current center of research (Figure 4B).

### 3.6 Keyword analysis

**FIGURE 5:**
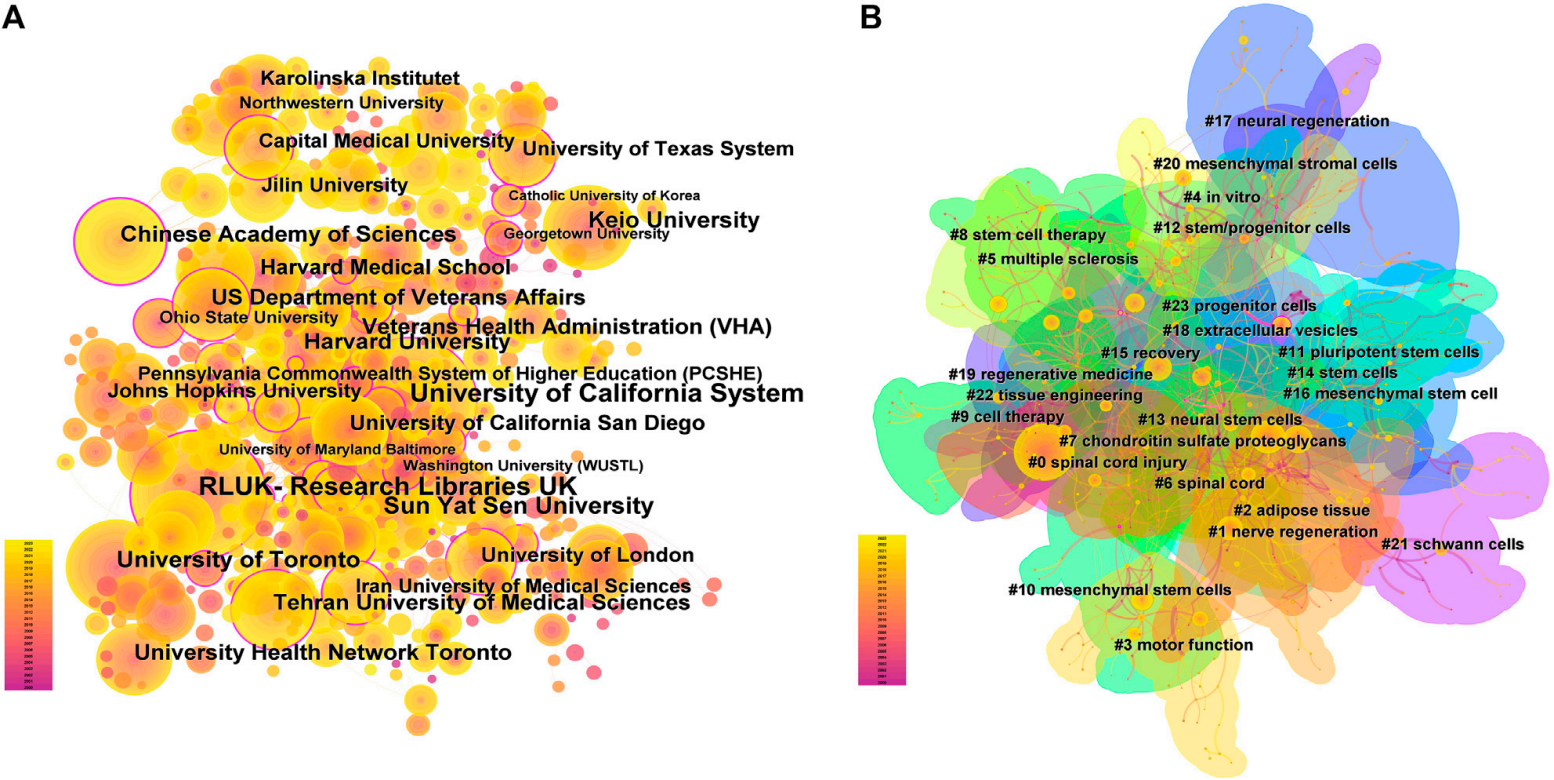
Keyword co-occurrence and clustering. **(A)** Co-occurrence analysis of keywords; **(B)** Clustering analysis of keyword.

**FIGURE 6:**
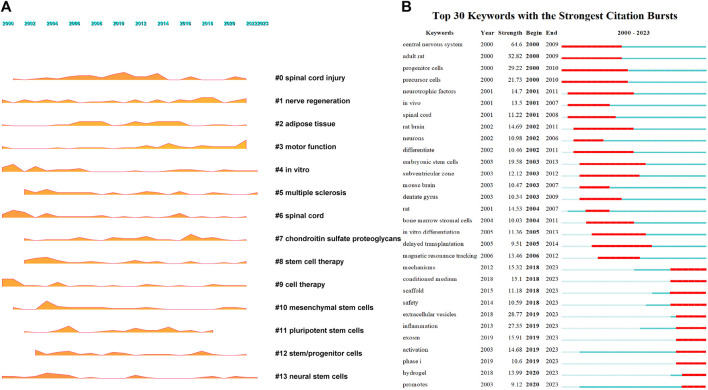
Keywords growth and decline and emergence [**(A)**. Keyword growth and decline, the larger the shadow area, the more important the keyword in a given year. **(B)**. Keywords emergence, red represents emergence, dark green represents the year in which the keyword appears, and light green represents the keyword does not appear].

After conducting co-occurrence analysis on 7,659 keywords, the top five keywords in terms of frequency were spinal cord injury, transplantation, stem cells, neural stem cells, and regeneration, see Figure 5A and Supplementary Table S5 for details. After clustering the keywords, 23 clusters were formed (Figure 5B), mainly focused on six aspects: spinal cord injury (clusters 0 and 6), neural regeneration (clusters 1, 17, and 19), stem cells (clusters 2, 8, 9, 10, 11, 12, 13, 14, 16, 20, 21, and 23), motor function recovery (clusters 3 and 15), extracellular vesicles (cluster 18), and tissue engineering (cluster 22). A landscape diagram was drawn to further explore the research trends in stem cell therapy for spinal cord injury. The results showed that stem cell therapy for spinal cord injury, neural regeneration, and motor function recovery have always been the research focus in this field and have received more extensive attention recently (Figure 6A). We then explored future development directions through keyword emergence analysis (keywords with a sharp increase in frequency). Thirty keywords with the highest emergence intensity were detected (Figure 6B). The top five keywords with the highest emergence intensity were central-nervous-system, adult rats, progenitor cells, extracellular vesicles, and inflammation, indicating that stem cell therapy, extracellular vesicle therapy, and inflammation are the current research foundation in spinal cord injury. The emergence of keywords related to molecular mechanisms, safety, extracellular vesicles, scaffolds, inflammation, clinical trials, and hydrogels has been persistent until 2023. This indicates that these keywords are current research priorities and will continue to be the focus of research in the field in the coming years.

## 4 Discussion

### 4.1 Research trend analysis

The number of publications to some extent represents the academic community’s attention and importance to the research field. From only 13 articles published in 2000 to an expected publication volume of 445 articles in 2023, stem cell therapy for spinal cord injury has received increasing attention from researchers and has accumulated 5544 publications. In general, the literature in this field is mainly published in a few highly specialized journals. There are 50 journals with a publication volume of more than 20 articles, accounting for 44.91% (2490/5544) of the total publications. These journals provide a good platform for research and academic exchange in stem cell therapy for spinal cord injury. Among them, Neural Regeneration Research has the highest publication volume and its growth rate is also the fastest. This indicates that this journal may become an important source of results and achievements in stem cell therapy for spinal cord injury in the future and should be given more attention.

### 4.2 Collaboration network analysis

Analyzing the collaboration between authors, institutions, and countries can not only reflect the publication volume but also visually reflect their connections, development, and status in the entire field, further revealing the structure and evolution of the discipline, and providing references for the development of stem cell therapy for spinal cord injury. Although there are many authors in this field, more than 70% of them have only participated in the publication of one article. This indicates that most authors have just entered this field and have not conducted in-depth research. It also demonstrates the enormous potential of this field, and over time, stem cell therapy for spinal cord injury will certainly see new breakthroughs. Although many academic groups have been formed in this field and the collaboration between authors within each group is relatively close, the collaboration network between different academic groups is relatively loose, and academic cooperation across regions and institutions has not been well established. Therefore, authors in this field should strengthen collaboration to promote the generation of significant academic achievements. Okano Hideyuki, Nakamura Masaya, and Fehlings Michael G, as the authors with the highest publication and citation numbers in this field, mainly focus on the molecular mechanisms of spinal cord injury and cell transplantation. For example, Professor Okano Hideyuki from Keio University has published several important papers exploring the molecular mechanisms of spinal cord injury and the potential of induced pluripotent stem cells for the treatment of spinal cord injury (Okada et al., 2006; Sasaki et al., 2009; Tsuji et al., 2010; Nori et al., 2011). These studies have played an important role in promoting the development of stem cell therapy for spinal cord injury. Xiao Zhifeng, Dai Jianwu, Zeng Yuanshan, and Sahib Seaab, as emerging academic groups in this field, are more concerned with the application of biomaterials in spinal cord injury (Han et al., 2015; Xu et al., 2023).Although 4020 institutions have participated in the publication of papers, and Sun Yat-sen University, the University of Toronto, and Keio University have the largest publication volumes, their academic influence is not as significant as that of institutions with relatively fewer publications, such as the University of California System, RLUK-Research Libraries United Kingdom, and the Chinese Academy of Sciences. In addition, although research papers from China are the most, China’s cooperation intensity with other countries is lower than that of the United States, and the current research center is not in China but in Europe. In fact, different countries should strengthen cooperation to better promote the generation of significant academic achievements and promote the development of this field. In conclusion, although stem cell therapy for spinal cord injury has received a lot of attention from many countries, institutions, and authors, the degree of communication and cooperation among them is not sufficient. It is urgent to eliminate academic barriers and strengthen collaboration and communication between different research institutions and teams (Li et al., 2021a; Ma et al., 2021).

### 4.3 Current research focus

The research hotspots and frontiers in a particular field have always been a hot topic for the academic community, as they directly reflect the most concentrated research questions and the latest research trends in a field. Keywords are usually highly condensed and concentrated summaries of the main content of an article, and keywords with high frequency are considered research hotspots. Aside from keywords related to spinal cord injury and stem cells, the most frequently appearing keywords are transplantation and regeneration, indicating that current research is mainly focused on stem cell transplantation and neural regeneration. Cluster analysis also shows that motor function recovery, nerve regeneration, stem cell transplantation, exosome and tissue engineering are the current research focus.

Spinal cord injury can lead to long-term bed rest due to motor dysfunction, as well as complications such as pulmonary infections, urinary tract infections and stones, pressure ulcers, deep vein thrombosis and pulmonary embolism, joint contractures, and heterotopic ossification (Jain et al., 2015; Ahuja et al., 2017a). Therefore, motor function recovery and neural regeneration are the key points of concern for researchers and patients. However, due to the unfavorable microenvironment formed in the injury area after spinal cord injury (Fan et al., 2018; Zheng et al., 2020; Bonizzato et al., 2021), the ongoing inflammatory response that leads to the formation of glial and fibrotic scar tissue in the injury site, and the limited intrinsic regenerative ability of nerve axons, spinal cord structure is difficult to spontaneously recover and sensory and motor dysfunction persists (Ahuja et al., 2017b; Fan et al., 2018; Anderson et al., 2022). Therefore, reshaping the microenvironment of spinal cord regeneration, promoting axonal and myelin regeneration, and ultimately rebuilding the damaged neural network are crucial for promoting motor function recovery after spinal cord injury. Although stem cell transplantation can mediate neural circuit rebuilding and functional improvement after spinal cord injury through various mechanisms such as promoting axonal regeneration, neuroprotection, and relay circuit formation, the specific molecular mechanisms and key target points for stem cell-mediated neural regeneration are not yet clear (Assinck et al., 2017). Moreover, spinal cord injury repair is an extremely complex process, and the newly formed neural circuit may not necessarily effectively improve motor function. Future research needs to further understand the pathological process after spinal cord injury, unravel the molecular mechanisms of neural regeneration, achieve functional neural circuit reconstruction, and improve motor function.

Although the results of preclinical studies on stem cell therapy are promising, there are still many issues that need to be addressed when transplanting stem cells into patients with spinal cord injury. The first issue is the problem of stem cell diffusion. Due to the presence of cerebrospinal fluid in the spinal cord and its constant flow, it is difficult for cells to settle in the injury site. This not only reduces the therapeutic effect of stem cells but also carries the risk of ectopic diffusion (Marsh et al., 2017). The second issue is the problem of stem cell directional differentiation, which is also the prerequisite for whether they can integrate with the host and form a functional network. Many studies have found that when neural stem cells are transplanted into non-neural areas such as the spinal cord of adult animals, they mainly differentiate into astrocytes, while very few differentiate into neurons. In addition, endogenous neural stem cells mainly differentiate into astrocytes rather than neurons at the site of spinal cord injury (Barnabe-Heider et al., 2010; Gilbert et al., 2022; Guo et al., 2022). Currently, at least 15 different tissue sources of stem cells have been applied in the treatment of spinal cord injury (Shang et al., 2022a), but the optimal tissue source, transplantation dose, transplantation timing and route, and number of transplantations are all unclear. These issues are crucial aspects that determine the translation of stem cell therapy from animal experiments to clinical applications and should be given priority attention.

In fact, simple stem cell transplantation alone is difficult to achieve directional differentiation into functional cells in the complex microenvironment of the spinal cord. Tissue engineering strategies need to be combined to synergistically promote neural regeneration after spinal cord injury. Tissue engineering strategies can optimize the combination of seed cells, scaffold materials, and bioactive factors according to the specific requirements of spinal cord repair, significantly improve the complex microenvironment changes after spinal cord injury, and induce endogenous/exogenous neural stem cells to differentiate into functional cells, thereby reshaping the neural regenerative microenvironment and repairing the damaged neural network, ultimately restoring motor function (Yang et al., 2015; Katoh et al., 2019; Liu et al., 2020; Zhu et al., 2021). Among them, tissue engineering strategies with biomaterials as the core provide a new direction for the treatment of spinal cord injury. Biomaterials can fill the cavities of the spinal cord injury site, load therapeutic drugs, induce differentiation of endogenous/exogenous neural stem cells into functional cells to bridge the severed spinal cord, improve the local microenvironment of the injury site, and promote functional recovery (Fuhrmann et al., 2017; Yang et al., 2020; Luo et al., 2022; Hu et al., 2023). Biomaterials combined with cytokines or stem cells can reduce the size of the injury area, diminish scar formation, promote neural regeneration, and restore motor function (Shrestha et al., 2014; Zhao et al., 2017a; Fuhrmann et al., 2017).

Recently, increasing evidence suggests that the therapeutic effect of stem cells is attributed to the paracrine mechanisms, mainly the function of extracellular vesicles, such as exosomes (Lai et al., 2010; Ren et al., 2020; Liu et al., 2021; Yuan et al., 2023). As nanoscale vesicles with a phospholipid bilayer, exosomes mediate intercellular communication, transferring specific information from parent cells to recipient cells, and thus affecting the recipient cells’ genotypic or phenotypic characteristics. Exosomes can promote spinal cord functional recovery, reduce the size of the spinal cord cavity, decrease apoptosis of damaged cells, reduce inflammatory responses, and promote angiogenesis and axonal regeneration (Huang et al., 2017; Sun et al., 2018; Yuan et al., 2019). The above functions of exosomes mainly rely on their containing various proteins, DNA, mRNAs, and microRNAs (miRNAs) from parent cells (Raposo and Stoorvogel, 2013; Kalluri and LeBleu, 2020). Compared with stem cells, exosomes have advantages of longer *in vivo* survival duration, lower carcinogenicity, and higher delivery efficiency (Huang et al., 2021). Exosomes are small vesicles with low potential for causing vascular blockage after intravenous injection, can cross the blood-brain barrier and enter the central nervous system, and can also deliver carefully selected miRNAs, siRNAs, and drugs through gene modification or as carriers (El Andaloussi et al., 2013; Lai et al., 2013; Long et al., 2017; Zhang et al., 2019). Exosomes cannot replicate *in vivo* and rapidly disintegrate after drug release; therefore, using exosomes for treatment is unlikely to cause tumors or malignant transformation (McCulloh et al., 2018). Kim et al. (2018) introduced oxidized nanoparticles into exosomes, which were then guided by a magnetic field to precisely target the desired area, greatly enhancing their targeting ability. In summary, exosomes have significant application prospects as both therapeutic agents and drug carriers, providing a new direction for the treatment of spinal cord injury. Although exosomes are intercellular communication molecules, the active mechanism of exosome therapy is still unclear. How exosomes penetrate the blood-brain barrier, how exosomes containing siRNAs specifically target the target cells, and which factors play a major role in the treatment of spinal cord injury are all unknown. Issues such as exosome heterogeneity, cell type specificity, purification, targeting, drug loading, and transport efficiency remain to be solved (Minciacchi et al., 2015; Pegtel and Gould, 2019).

In conclusion, neural regeneration, motor function recovery, stem cell transplantation, exosomes, and tissue engineering are the five main research areas for stem cell therapy in spinal cord injury. The results of landscape analysis also support this, with research on stem cell therapy, neural regeneration, and motor function recovery consistently increasing since 2000.

### 4.4 Future research directions

Exploring future research directions for stem cell therapy in spinal cord injury through keyword emergence analysis. Although the keywords with the highest emergence intensity are “adult rats, extracellular vesicles, and inflammation,” the emergence of molecular mechanisms, inflammation, safety, clinical trials, extracellular vesicles, scaffolds, and hydrogels in stem cell therapy for spinal cord injury has continued until 2023, indicating that these aspects are the focus of attention in this field in the coming years.

Firstly, the molecular mechanism by which stem cells exert their effects has been extensively studied, but the key targets/pathways are still unclear, and the interactions between different targets/pathways remain unknown. Especially for the recently discovered extracellular vesicles secreted by stem cells, it is unclear whether they can replace stem cells to exert their functions. Therefore, the molecular mechanisms of stem cells and their extracellular vesicles in the treatment of spinal cord injury, especially the role of inflammation in this process, remain a hot research topic for the future.

Secondly, as we mentioned earlier, tissue engineering strategies offer tremendous prospects for treating spinal cord injury. In the tissue engineering repair of spinal cord injury, the main role of a biomaterial scaffold is to provide physical bridging and guidance functions, as well as serving as a carrier for seed cells or bioactive factors (Yi et al., 2019; Li et al., 2021b).Currently, the most common types of scaffolds are three-dimensional scaffolds, single/multiple-channel conduits, and hydrogels, which can provide support for neural regeneration. In recent years, there has been a growing research and application of hydrogels. Hydrogels are a class of highly hydrophilic three-dimensional network structures that rapidly swell in water and maintain a large volume of water, with extremely high flexibility (Wang et al., 2022).The structure of spinal cord tissue is similar to that of hydrogels (Dumont et al., 2019).Hydrogels that have a matched elastic modulus as the spinal cord, especially injectable hydrogels, have the advantages of non-invasive or minimally invasive implantation, avoiding the risks of large-scale surgery, and the gel formed *in situ* easily adheres to the irregular spinal cord, thus achieving an excellent implant-host spinal cord interface (Taylor and Sakiyama-Elbert, 2006; Chockalingam et al., 2007; Yan et al., 2023).Hydrogels can be used as carriers for cell or cytokine delivery or can directly stimulate axon growth and tissue repair (Dumont et al., 2019). With the development of biological tissue engineering technology, especially the progress in 3D bioprinting organ reconstruction technology, the use of hydrogels as bio-ink to organically combine seed cells, bioactive factors, and other components is expected to construct highly biomimetic artificial spinal cord tissue (Dutta et al., 2021). Especially in recent years, the effect of exosomes in the repair of spinal cord injury has also received widespread attention, and in future research, adding exosomes to hydrogels will be a research hotspot. For example, Gao et al. assembled exosomes derived from stem cells in a peptide-modified hydrogel for transplantation, enabling the implanted exosomes to demonstrate effective retention and sustained release in host neural tissues, thereby inducing effective relief of the microenvironment in spinal cord injury (Li et al., 2020). Although significant progress has been made in spinal cord injury repair research using biomaterial scaffolds, such as hydrogels as representatives, the clinical application of biomaterial scaffold transplantation in spinal cord injury still has a long way to go. The mechanism by which the performance of biomaterial scaffolds itself regulates cell behavior and the microenvironment is still unclear, further elucidation of mechanisms such as neural regulation and repair is needed to lay a solid foundation for the clinical translation of biomaterials in spinal cord injury. From the perspective of translation, considering the repair strategy starting from the regenerative environment inside the patient’s body, while simultaneously taking into account the hardness, conductivity, drug release, and other factors of the biomaterial, may increase the likelihood of tissue engineering success. Although the biodegradable material scaffold represented by hydrogels has made great progress in spinal cord injury repair research, there are still many challenges. How to replicate the function of natural extracellular matrix using a biomaterial scaffold and how to control the material preparation process, including the regulation of its biomechanics, modulus, porous structure, micro/nanostructure, and their impact on cell behavior, are still the focus of future research (Tay et al., 2011; Carotenuto et al., 2022). In conclusion, biomaterials, as the core of tissue engineering regenerative repair, combined with cell, bioactive factors, drugs and other components to regulate the spinal cord injury regeneration microenvironment, rebuild the neural functional circuit, are the core contents of future spinal cord injury repair research.

Finally, clinical translation of stem cell therapy and its safety issues have yet to be resolved. As our keyword emergence results show, clinical trials have become a key topic of focus in this field in recent years. The main reason is the positive results of stem cell therapy in animal models of spinal cord injury and the successful application in other diseases, such as malignant tumors of the hematopoietic system, burns, and corneal transplants, which have accelerated the process of clinical translation (Chhabra et al., 2019; De Luca et al., 2019; Shang et al., 2022a). Scholars from various countries are actively conducting clinical trials for spinal cord injury treatment using stem cells, and have initially demonstrated the effectiveness of stem cell transplantation in treating spinal cord injury (Xiao et al., 2016; Zhao et al., 2017b). However, there is high heterogeneity among different studies, such as different patient types, inconsistent spinal cord injury segments, varying content and duration of subsequent rehabilitation training, and a limited number of subjects included in clinical trials. At present, there is a lack of unified standards for staging spinal cord injury, a lack of standardized transplantation strategies for stem cells, inconsistent assessment methods, a lack of sufficient controls, double-blind trials, *etc.*, which have led to unclear clinical efficacy of stem cells and difficulty in large-scale promotion to more patients (Yoon et al., 2007; Zholudeva and Lane, 2019). In fact, safety should be the first consideration in the clinical translation of stem cell therapy. Stem cell transplantation may cause 28 adverse reactions such as neuropathic pain, abnormal feeling, muscle spasms, vomiting, urinary tract infection, and may be involved in tumor formation (Andrews et al., 2022; Shang et al., 2022b; Shamsian et al., 2022).In addition, ethical issues, regulatory controversies, carcinogenesis, cell autophagy, virus transmission, and other risks associated with stem cell transplantation have yet to be resolved (Cyranoski, 2013; Shang et al., 2022b). As shown in Shang et al.'s study (Shang et al., 2022b), the clinical translation of stem cell therapy for spinal cord injury is still immature. Therefore, future preclinical research should focus more on the safety issues of stem cells and the transplantation process, as well as the optimal transplantation strategy for stem cells, in order to improve the therapeutic effects of stem cell repair in spinal cord injury. Of course, future clinical trials should carefully recruit patients, design experiments, measure results, analyze data, and report findings, ensuring patient safety. By doing so, the safety and effectiveness of stem cell therapy can be fully explored, benefiting more patients with spinal cord injury.

Spinal cord injury regeneration and repair has always been a global challenge that has troubled the medical community, as well as a complex biological process. Nervous tissue contains a large number of different types of cells, in addition to the invasion of the lymphatic system after injury, as well as the activation of various types of endogenous stem cells, increasing the heterogeneity within the cell population (Varadarajan et al., 2022; Brunet et al., 2023). In future research, it will be necessary to elucidate the molecular mechanisms of spinal cord injury and repair, identify key molecules associated with spinal cord tissue regeneration, and reconstruct a microenvironment conducive to neural regeneration through functional scaffold materials combining biomaterials, growth factors, and stem cells. Through the application of high-throughput sequencing concepts and techniques, gradually transitioning from previous static observations and single mechanism analysis to multi-mechanism collaborative dynamic monitoring of the entire disease progression analysis, exploring the molecular mechanisms associated with spinal cord injury at different stages (Wang et al., 2022; Li et al., 2022). Elucidating the mechanisms of endogenous and exogenous stem cell activation, migration, directed neuron differentiation, and connection with host neurons after spinal cord injury, fundamentally overcoming the bottleneck of existing stem cell therapy. In addition, it is necessary to strengthen the clinical translation research of spinal cord injury regeneration and repair, establish a clinical research plan for preoperative diagnosis, surgical plan, postoperative functional evaluation, and integrated rehabilitation of spinal cord injury, and promote the rapid development of basic research and clinical translation of spinal cord injury.

In summary, restoring the conduction pathway of neural regeneration and promoting the reconstruction of functional connections between the injury site and target area are still the basis and key to functional recovery from spinal cord injury. The microenvironment of the injury site is extremely complex, posing significant challenges to repair. Although stem cells have brought hope to the repair of spinal cord injury, the mechanisms, transplantation strategies, and safety of stem cells need more attention. Extracellular vesicles derived from stem cell sources and tissue engineering strategies with biomaterials as the core also offer new directions for stem cell therapy for spinal cord injury.

### 4.5 Limitations

This study employed the method of bibliometrics to quantitatively analyze the current status and Frontier hotspots of stem cell therapy for spinal cord injury. The findings of this study provide a foundation for future in-depth research in this field. Nevertheless, it is important to acknowledge that this study is not without limitations. Firstly, by only including journal articles and excluding comments and other types of literature, some popular research topics may have been missed. Secondly, excluding non-English articles may have an impact on the conclusion. In addition, we only included data from the WoSCC database, while ignoring other databases such as PubMed, Scopus, Embase, which may overlook some research. However, WoSCC is the most commonly used database for bibliometric analysis (Yeung et al., 2020; Wu et al., 2021a; Wu et al., 2021b).The data from WoSCC is sufficient to reflect the current status of research on stem cell therapy for spinal cord injury. Additionally, different databases have differences in file export formats and merging databases is not always the best option.

## 5 Conclusion

Stem cell therapy for spinal cord injury has attracted increasing attention, but the collaboration intensity between different authors, institutions, and countries in this field needs to be strengthened. More importantly, the key target/molecular mechanisms for stem cell repair of spinal cord injury, transplantation strategies (dose, timing, route, source, frequency), clinical translation of stem cells, safety, tissue engineering strategies, and the therapeutic potential of exosome have not been clearly addressed. These aspects are the key issues that should be focused on and urgently resolved in this field, and they represent the future research directions.

## Data Availability

The original contributions presented in the study are included in the article/Supplementary Material, further inquiries can be directed to the corresponding author.
