# Supplementary material for: Bibliometric analysis of stem cells for spinal cord injury: current status and emerging frontiers
Source: Front Pharmacol. 2023 Jul 18;14:1235324. doi: 10.3389/fphar.2023.1235324 (PMC10392836; doi:10.3389/fphar.2023.1235324)
Supplement: Supplementary file 1 [file Table1.DOCX]

**Bibliometric Analysis of Stem Cells for Spinal Cord Injury: Current Status and Emerging Frontiers**

Zhizhong Shang^1^, Pingping Wanyan^2,3^, Mingchuan Wang^1^, Baolin Zhang^1^, Xiaoqian Cui^1^, Xin Wang^1,4,5*^

1. The First Clinical Medical College of Lanzhou University, Lanzhou, 730000, China

2. Gansu University of Chinese Medicine, Lanzhou, 730000, China

3. The Second Hospital of Lanzhou University, Lanzhou, 730000, China

4. Chengren Institute of Traditional Chinese Medicine, Gansu Province, 730000, China

5. Department of Spine, Changzheng Hospital, Naval Medical University, Shanghai, 200003, China

*Corresponding author:

E-mail: wangxinldyy@126.com (Xin Wang)

Full postal address: Lanzhou University, Donggang West Road No 199, Chengguan District, Lanzhou City, 730030, People’s Republic of China.

**Table 1: Top 10 journals by cumulative publication growth**

| Year | NEURAL REGENERATION RESEARCH | CELL TRANSPLANTATION | EXPERIMENTAL NEUROLOGY | JOURNAL OF NEUROTRAUMA | PLOS ONE | INTERNATIONAL JOURNAL OF MOLECULAR SCIENCES | BIOMATERIALS | STEM CELL RESEARCH & THERAPY | JOURNAL OF NEUROSCIENCE | BRAIN RESEARCH |
| --- | --- | --- | --- | --- | --- | --- | --- | --- | --- | --- |
| 2000 | 0 | 1 | 1 | 0 | 0 | 0 | 0 | 0 | 0 | 0 |
| 2001 | 0 | 1 | 1 | 1 | 0 | 0 | 0 | 0 | 1 | 1 |
| 2002 | 0 | 3 | 2 | 2 | 0 | 0 | 0 | 0 | 1 | 1 |
| 2003 | 0 | 3 | 4 | 3 | 0 | 0 | 0 | 0 | 3 | 1 |
| 2004 | 0 | 4 | 11 | 8 | 0 | 0 | 0 | 0 | 3 | 1 |
| 2005 | 0 | 5 | 20 | 10 | 0 | 0 | 0 | 0 | 6 | 6 |
| 2006 | 0 | 7 | 29 | 20 | 0 | 0 | 2 | 0 | 13 | 8 |
| 2007 | 0 | 12 | 31 | 26 | 0 | 0 | 4 | 0 | 17 | 9 |
| 2008 | 3 | 18 | 36 | 27 | 2 | 0 | 4 | 0 | 22 | 11 |
| 2009 | 8 | 24 | 41 | 39 | 6 | 0 | 8 | 0 | 26 | 16 |
| 2010 | 18 | 27 | 48 | 41 | 11 | 0 | 14 | 1 | 31 | 23 |
| 2011 | 25 | 40 | 55 | 51 | 25 | 1 | 16 | 3 | 36 | 28 |
| 2012 | 35 | 54 | 61 | 56 | 39 | 2 | 24 | 4 | 39 | 31 |
| 2013 | 46 | 68 | 68 | 59 | 52 | 4 | 29 | 9 | 45 | 34 |
| 2014 | 63 | 77 | 80 | 62 | 69 | 6 | 36 | 12 | 48 | 35 |
| 2015 | 80 | 90 | 88 | 65 | 81 | 10 | 39 | 23 | 52 | 44 |
| 2016 | 93 | 102 | 97 | 68 | 86 | 16 | 46 | 25 | 56 | 52 |
| 2017 | 102 | 107 | 105 | 73 | 91 | 22 | 52 | 25 | 60 | 56 |
| 2018 | 111 | 114 | 107 | 82 | 93 | 31 | 61 | 32 | 64 | 59 |
| 2019 | 119 | 124 | 113 | 92 | 93 | 40 | 65 | 42 | 64 | 63 |
| 2020 | 129 | 129 | 117 | 95 | 96 | 51 | 71 | 50 | 67 | 66 |
| 2021 | 137 | 134 | 123 | 98 | 96 | 68 | 79 | 68 | 68 | 67 |
| 2022 | 150 | 139 | 127 | 101 | 101 | 82 | 82 | 76 | 69 | 67 |
| 2023 | 162 | 141 | 129 | 102 | 102 | 90 | 85 | 79 | 69 | 67 |

**Table 2: Institutions with the top 10 volume of publications**

| **Rank** | **Affiliation** | **Articles** |
| --- | --- | --- |
| 1 | SUN YAT SEN UNIV | 420 |
| 2 | UNIV TORONTO | 303 |
| 3 | KEIO UNIV | 296 |
| 4 | UNIV TEHRAN MED SCI | 250 |
| 5 | ZHEJIANG UNIV | 196 |
| 6 | UNIV CALIF IRVINE | 164 |
| 7 | UNIV MIAMI | 156 |
| 8 | IRAN UNIV MED SCI | 148 |
| 9 | NANTONG UNIV | 145 |
| 10 | YONSEI UNIV | 145 |

**Table 3: The top 10 institutions in terms of intermediary centrality**

| **Rank** | **Institutions** | **Betweenness centrality** |
| --- | --- | --- |
| 1 | RLUK- Research Libraries UK | 0.23 |
| 2 | University of California System | 0.18 |
| 3 | Harvard University | 0.1 |
| 4 | State University System of Florida | 0.08 |
| 5 | Tehran University of Medical Sciences | 0.08 |
| 6 | Capital Medical University | 0.07 |
| 7 | Harvard Medical School | 0.06 |
| 8 | Institut National de la Sante et de la Recherche Medicale (Inserm) | 0.06 |
| 9 | National Yang Ming Chiao Tung University | 0.06 |
| 10 | Pennsylvania Commonwealth System of Higher Education (PCSHE) | 0.06 |

**Table 4: Publication volumes of different countries**

| **Rank** | **Country** | **Frequency** | **Rank** | **Country** | **Frequency** |
| --- | --- | --- | --- | --- | --- |
| 1 | CHINA | 5357 | 36 | MALAYSIA | 36 |
| 2 | USA | 4266 | 37 | CHILE | 35 |
| 3 | JAPAN | 1370 | 38 | GREECE | 32 |
| 4 | IRAN | 1224 | 39 | FINLAND | 31 |
| 5 | CANADA | 924 | 40 | DENMARK | 28 |
| 6 | SOUTH KOREA | 882 | 41 | NORWAY | 24 |
| 7 | UK | 572 | 42 | JORDAN | 22 |
| 8 | GERMANY | 539 | 43 | HUNGARY | 21 |
| 9 | ITALY | 499 | 44 | NEW ZEALAND | 16 |
| 10 | SPAIN | 406 | 45 | IRAQ | 13 |
| 11 | AUSTRALIA | 367 | 46 | CROATIA | 12 |
| 12 | INDIA | 282 | 47 | URUGUAY | 12 |
| 13 | BRAZIL | 279 | 48 | THAILAND | 11 |
| 14 | CZECH REPUBLIC | 259 | 49 | UKRAINE | 11 |
| 15 | SWEDEN | 225 | 50 | PAKISTAN | 9 |
| 16 | FRANCE | 213 | 51 | BANGLADESH | 6 |
| 17 | TURKEY | 194 | 52 | NIGERIA | 6 |
| 18 | PORTUGAL | 165 | 53 | SOUTH AFRICA | 6 |
| 19 | RUSSIA | 160 | 54 | COLOMBIA | 5 |
| 20 | POLAND | 143 | 55 | LEBANON | 5 |
| 21 | BELGIUM | 128 | 56 | BULGARIA | 4 |
| 22 | SWITZERLAND | 119 | 57 | GEORGIA | 4 |
| 23 | EGYPT | 111 | 58 | QATAR | 4 |
| 24 | SINGAPORE | 100 | 59 | GHANA | 3 |
| 25 | AUSTRIA | 99 | 60 | VIETNAM | 3 |
| 26 | NETHERLANDS | 99 | 61 | ECUADOR | 2 |
| 27 | MEXICO | 97 | 62 | KUWAIT | 2 |
| 28 | SLOVAKIA | 80 | 63 | LITHUANIA | 2 |
| 29 | ISRAEL | 79 | 64 | ALGERIA | 1 |
| 30 | ROMANIA | 77 | 65 | BELARUS | 1 |
| 31 | IRELAND | 63 | 66 | COSTA RICA | 1 |
| 32 | ARGENTINA | 45 | 67 | CYPRUS | 1 |
| 33 | SAUDI ARABIA | 45 | 68 | LIBYA | 1 |
| 34 | INDONESIA | 42 | 69 | PHILIPPINES | 1 |
| 35 | SERBIA | 37 | 70 | SAN MARINO | 1 |

**Table 5: The top 10 keywords by frequency of appearance**

| **Keyword** | **Occurrences** |
| --- | --- |
| Spinal cord injury | 1890 |
| Transplantation | 1030 |
| Stem cells | 949 |
| Neural stem cells | 590 |
| Regeneration | 588 |
| Expression | 556 |
| Recovery | 485 |
| Bone marrow | 453 |
| Axonal regeneration | 436 |
| Stromal cells | 425 |
